# On Union by the First Intention

**Published:** 1829-07-01

**Authors:** 


### XXVI.

### M. M. DELMAS AND TAVERNIER, ON UNION BY THE FIRST INTENTION.

It appears that union by the first intention is far from being yet universally adopted in France, though many of the leading surgeons are ranked as its supporters. There, as here, science, like every thing else, has its Whigs and its Tories, its sticklers for "the wisdom of their ancestors," and its mad liberals raging for innovation, and respecting no wisdom but their own. We are beginning, in this country, to discover that the *ultras* on both sides are wrong, and our views of union by the first intention not being of quite such recent growth as amongst our lively neighbours, have softened by the sobering influence of time into a more staid but stable form. In fact, our most eminent surgeons, though fully alive to the great advantages of union by the first intention, in the great majority of wounds, whether caused by accident or secundum artem, are yet not blind to the fact, that it frequently is, and has been attempted, without any reasonable chance of success, and therefore, with highly injurious effects. It is not our intention to occupy, like M.M. Delmas and Tavernier, some *thirty* pages in *arguing* the question, but merely to extract the heads of a case or two.

CASE 1. *Amputation of the Breast—complete union in nine days—subsequent Cancer of the Bones.*

The breast of a lady fifty years of age, affected with open cancer, was removed by the father of M. Delmas, in the month of September, 1819. Some axillary glands were also removed, and the wound was of very great size. Three ligatures were applied, and after waiting for some little time, and sponging the wound with warm water, to see whether other vessels would bleed, the lips of the wound were brought together. Next day some of the straps were removed, and more than an inch at each angle of the wound had united. On the 7th day the ligatures had all come away, and on the 9th, the wound was completely healed.

In another patient of the same surgeon, the recovery was so rapid that she returned to her work as a servant on the 8th day. To return to the subject of the present case, she died in two years after the operation with symptoms of inflammation of the chest, but both before and after death the bones broke with the slightest force. If the poor lady leaned upon her hand to change her position, a fracture was the consequence, and the persons employed about the corpse, heard the bones snap in every direction on moving her into the coffin, &c.

This case, briefly as we have given it, illustrates an important point with regard to the operation for cancer of the breast, viz. that although it may ultimately fail, it gives the patient an additional lease of life, and generally kills at last by *internal* disease, saving her the misery of *seeing* herself sink with a foul, painful, and disgusting sore. Perhaps we may be allowed to make a remark on the management of the bleeding vessels in this operation. The best surgeons of the present day make it a point to search for and secure every vessel, even of moderate size, which bleeds, or is likely to do so. This, though laughed at by some *bold* practitioners, is an excellent precaution, as it totally prevents the risk of secondary hæmorrhage, one of the most unfortunate accidents that can occur in operations of this description.

Case 2. *Compound Dislocation of the Ankle-joint—Compound Fracture of the Leg—Traumatic Delirium—Amputation—Successful Issue.*

Madame Clavieres, ætatis fifty-five, whilst walking on a bank five or six feet high, slipped, and came with the whole weight of her body (an enormously fat one) on the right foot. She was carried to her house, where she was immediately seen by Dr Bonnemaison, and shortly afterwards by M. Delmas and his father. The foot was strongly twisted inwards, bruised and torn; the integuments over the outer ankle were lacerated to the extent of two inches, and through the laceration protruded about an inch of the lower extremity of the fibula; and lastly, it was evident that the ligaments uniting the tibia behind to the astragalus and os calcis were torn. About the lower third of the leg was another laceration of the soft parts through which the fibula also projected, and the tibia was fractured at the same place. Several other bruises existed on the leg. The superior extremities were agitated with spasmodic contractions—the cries were incessant—the eyes were fixed—the respiration hurried—the pulse hard and small—the patient incoherent. Owing to the absence of other medical men the operation was deferred for four or five hours, when it was performed about three inches below the upper extremity of the tibia. The leg was of such an uncommon size, that the incision was made through skin, muscle, and all to the bone, instead of attempting to separate the integuments from the muscles, as usual. On examination of the limb, a great quantity of blood was extravasated in different parts, the muscles were greatly injured, of brown colour, and not adhering to the bones, the fracture of the fibula was oblique, the articulation of the tibia and astragalus laid open, that of the fibula entirely destroyed.

Antispasmodics were administered, and next day the delirium had entirely subsided. At the end of four days a third of the stump had united, but owing to the enormous size of the wound, it was five or six weeks before the patient was completely recovered.

We believe that few cases occur of so eminently successful a nature as the foregoing, certainly very few or none in the hospital practice of a large town. The delirium which followed so hard upon the accident was evidently of the traumatic kind, and its subsidence after the operation will afford a precedent, and assist the surgeon in deciding on amputation in a future case. We have seen one or two operations performed whilst the patient was labouring under incoherence, although not actual delirium, but they all proved fatal. It will be observed, that the two cases we have related, have been given by their author with different views, but it matters not. If facts can be turned to account, it signifies little with what particular *object* they were put forth.

